# Recurrence of Goodpasture syndrome without circulating anti-glomerular basement membrane antibodies after kidney transplant, a case report

**DOI:** 10.1186/s12882-018-1197-6

**Published:** 2019-01-08

**Authors:** V. Thibaud, N. Rioux-Leclercq, C. Vigneau, S. Morice

**Affiliations:** 1 Department of Hematology, CHU Rennes, Rennes, France; 2 Department of Pathology, CHU Rennes, Rennes, France; 3 Department of Nephrology, CHU Rennes, Rennes, France

**Keywords:** Kidney transplant, Goodpasture syndrome (GS), Anti-glomerular basement membrane (GBM) disease, End-stage renal disease (ESRD)

## Abstract

**Background:**

Goodpasture Syndrome (GS) is an autoimmune disease caused by the development of auto-antibodies against the Glomerular Basement Membrane (GBM). Linear deposit of immunoglobulins G on the GBM detected by immunofluorescence analysis of renal biopsies is a GS pathognomonic finding. GS is commonly monophasic and its incidence is 1.6 case per million per year.

**Case presentation:**

This report describes and discusses the case of a 40-year-old woman who one year after allograft kidney transplant, presented with acute pulmonary and renal symptoms of GS, leading to acute graft dysfunction, without circulating anti-GBM antibody detection in laboratory assays. She received a living donor kidney transplant 4 years after the first diagnosis of GS without circulating anti-GBM antibodies, when considered in remission.

**Conclusions:**

In both episodes, the diagnosis of GS was based exclusively on the kidney biopsy that showed rapidly progressing glomerulonephritis with deposition of immunoglobulins G on the GBM. Although rare, the management of patients with GS without circulating anti-GBM antibodies is difficult due to the lack of standardized follow-up guidelines to reduce the risk of GS recurrence after kidney transplantation.

## Background

Goodpasture Syndrome (GS) is an autoimmune disease mediated by anti-Glomerular Basement Membrane (GBM) antibodies the first description of which was attributed to Ernest Goodpasture [1]. The linear immunofluorescence staining for immunoglobulin G (IgG) on the GBM in kidney biopsy specimens is a pathognomonic finding of GS. This syndrome is characterized by the presence of Rapidly Progressive GlomeruloNephritis (RPGN) that leads to acute renal failure, and of potentially life-threatening pulmonary hemorrhages [2]. GS is commonly described as a monophasic illness. Although, its incidence has been recently estimated at 1.6 case per million per year [3], it accounts for approximately 20% of all RPGN cases. The titer of circulating antibodies against collagen type IV, alpha-3 [4] is considered a measure of disease severity and correlates with the renal outcomes [5]. It may also be a predictive factor of relapse. Moreover, Anti-Neutrophil Cytoplasmic Antibodies (ANCA) with affinity for myeloperoxidase are detected in 25% of patients with GS. Circulating antibodies are undetectable in about 5% of patients with GS [6].

Here, we describe and discuss the case of a woman with a GS relapse without detectable circulating anti-GBM antibodies that led to acute renal allograft dysfunction one year after transplantation. The graft was performed 4 years after the first diagnosis of GS without circulating anti-GBM antibodies (in 2011), when the patient was considered in remission.

## Case presentation

On 16 December 2011, a 40-year-old white woman was hospitalized with dyspnea and a small-volume hemoptysis that had started 2 weeks before. She reported asthenia, but no weight loss, cigarette smoking (20 pack-years) that was not stopped afterwards, no exposure to toxic chemicals. Her medical history included pre-eclampsia during her two pregnancies, but no previous pulmonary disease or family history of renal/cardiac/pulmonary diseases. No other relevant finding was recorded.

Clinical examination upon admission highlighted apyrexia, hypertension (184/105 mmHg), pulse rate of 96 beats/minute, and skin pallor. A chest X-ray showed bilateral infiltrates, and the thoracic CT scan indicated diffuse and bilateral ground-glass opacification. The laboratory work-up showed normocytic normochromic anemia (hemoglobin level of 7 g/dL), but normal platelet and leucocyte counts. The creatinine level of 614 μmol/L (50 μmol/L in June 2011) indicated acute renal failure. Due to respiratory failure and renal impairment, the patients received three daily boluses of methylprednisolone (500 mg) followed by 1 mg/kg/day of prednisone.

A bronchoscopy performed on day 4 after hospitalization revealed the presence of hematic traces with a Golde score of 197 (bacterial cultures were negative). Serologic tests for auto-antibodies (antinuclear antibodies, ANCA, and anti-GBM antibodies) were negative, and the hemolytic complement fractions within the normal values (C3 = 1.22 g/L and C4 = 0.28 g/L). The ELISA test for anti-GBM antibodies using purified collagen IV alpha3 chain was negative. The renal biopsy showed fibrinoid necrosis in 10 glomeruli (among the 29 assessed; 34.5%), glomerulosclerosis in 30% of glomeruli, and cellular glomerular crescents in 28%. Immunofluorescence analysis revealed linear deposition of IgG, compatible with GS.

The patient underwent daily PLasmatic EXchanges (PLEX) for 11 days and started oral immunosuppressive therapy (100 mg of cyclophosphamide per day) on day 13 of the prednisone treatment. Due to severe renal failure and anuria, hemodialysis (3 times per week) was started on December 20, 2011. Hemoptysis stopped rapidly, but diuresis was not improved. At day 37 of hospitalization, due to neutropenia (<1G/L), the cyclophosphamide treatment was reduced to 75 mg per day, and then discontinued after 3 months. The patient remained on dialysis. As the serologic tests were all negative in 2011, the anti-GBM antibodies could not be monitored, but the patient did not show any other GS symptom in the following years.

In 2014, when the patient was still taking 5 mg of prednisone per day, a new episode of hemoptysis occurred confirmed by bronchoscopy. This was associated with acute pneumonia of the left lung lower lobe, with favorable outcome after treatment with prednisone (50 mg per day for 1 week) and fluoroquinolone-based antibiotic therapy.

In March 2015, the patient received a living donor (her mother) kidney transplant. In the years from the GS episode to the kidney transplant, all serologic tests for auto-antibodies were negative. Conversely, panel-reactive antibodies against class I and class II antigens were detected, but not against the donor’s human leukocyte antigen (HLA) (identical HLA profiles for donor and patient). Anti-Epstein Barr virus and cytomegalovirus IgGs were detected in serum samples from donor and recipient. The patient was induced with anti-thymocyte globulin (rabbit) and received standard immunosuppressive therapy with mycophenolate mofetil (MMF), tacrolimus (residual serum tacrolimus between 6 and 8 ng/mL), and prednisone. After the graft, the creatinine level was stabilized between 110 and 130 μmol/L. She developed new onset diabetes after transplantation that was treated with metformin and repaglinide.

**Fig. 1:**
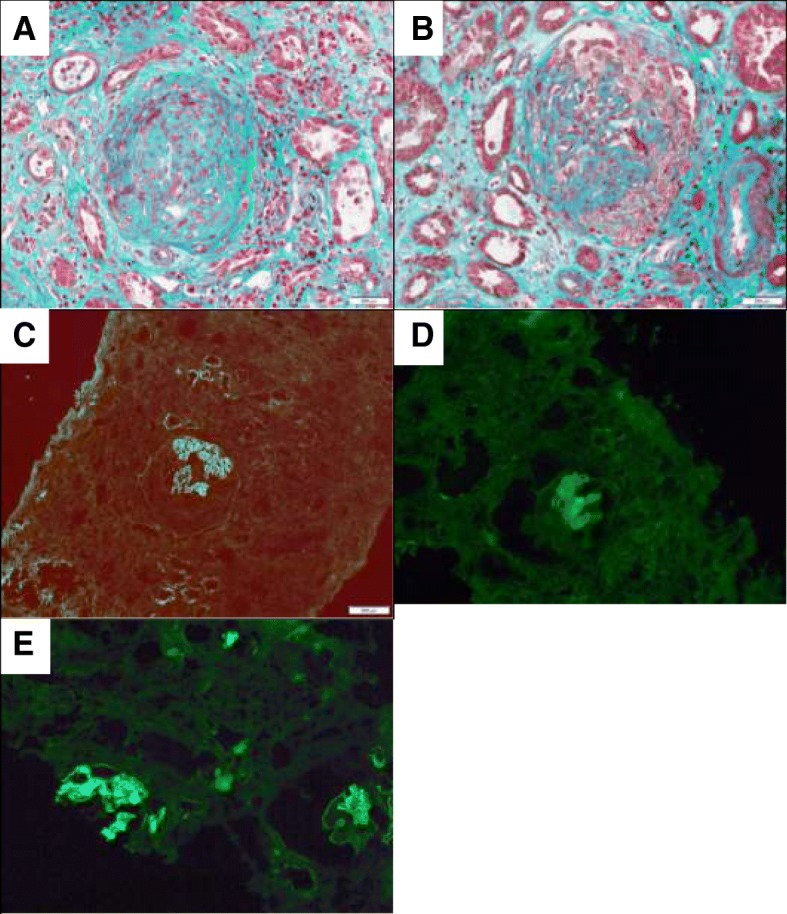
Renal biopsy showing cellular glomerular crescents and linear IgG deposition on the glomerular basement membrane (GBM). Panel **a**: Fibrocellular glomerular crescents with focal necrosis, Panel **b**: Glomeruli with semi-circumferential extracapillary crescents and segmental necrosis, Panel **c**: Immunofluorescence analysis to detect immunoglobulins G (IgG) on the GBM, Panel **d**: Immunofluorescence analysis to detect IgG1 on the GBM, Panel **e**: Immunofluorescence analysis to detect IgG4 on the GBM

In November 2016, microscopic hematuria without any proteinuria or renal dysfunction (creatinine level = 104 μmol/L) was detected. In February 2017, the patient was hospitalized because of hemoptysis and anuric acute renal failure (creatinine level = 1696 μmol/L). Like in 2011, blood pressure was increased (220/113 mmHg). The laboratory work-up showed normocytic normochromic anemia (hemoglobin level of 10.4 g/dL), platelet count of 100G/L, and normal leucocyte levels. At this time, the patient was taking tacrolimus (5 mg per day; residual tacrolimus level = 4.2 ng/mL), MMF (500 mg twice per day), and prednisone (5 mg per day). The serologic tests for auto-antibodies (antinuclear antibodies, ANCA, and anti-GBM antibodies) were negative, and the hemolytic complement factors within normal levels (CH50 = 79 U/mL, C3 = 1.28 g/L and C4 = 0.35 g/L). Renal biopsy of the transplanted kidney (25 glomeruli) confirmed the GS relapse with glomerulosclerosis in 36% of the analyzed glomeruli, cellular glomerular crescents in 56%, and linear IgG deposition on the GBM (Fig. 1).

The patient underwent daily PLEX for 14 days, and received prednisone (500 mg/day for the first 3 days followed by 1 mg/kg/day). At the end of the 14th PLEX, a bronchoscopy indicated active hemoptysis with the presence of 99.5% sideroblasts and a Golde score of 200. Due to the absence of effect, PLEX was stopped and intravenous infusion of cyclophosphamide (500 mg/m^2^ every 21 days) was introduced combined with prednisone (5 mg per day) and tacrolimus (2 mg twice per day).

After five infusions of cyclophosphamide the patient has now recovered and the anemia is under control with EPO supplementation. Conversely, the grafted kidney does not work, and the patient needs hemodialysis 3 times per week. Tacrolimus has been stopped.

## Discussion and conclusions

Relapse is a rare event in GS and there is only a limited number of reported cases in the literature [2, 7–13]. This reflects the usually self-limited nature of auto-antibody formation in this disease. Histological GS relapse in kidney transplants has been reported in nearly 50% of patients, if the graft is done in the presence of serum anti-GBM antibodies [14]. For this reason, the current guidelines recommend serum anti-GBM antibody negative results for at least 12 months before kidney transplantation. When these guidelines are followed, GS relapse in transplanted kidneys is lower than 5% [15].

This case report highlights the complex management of patients with biopsy-proven GS and without serum anti-GBM antibodies for whom no guideline is available.

More than 20 years ago, it was shown that pathogenic auto-antibodies are predominantly directed against the non-collagenous domain of type IV collagen epitopes, mostly in a peptide sequence of the alpha-3 chain [4]. These epitopes are used in the most common assays to detect circulating anti-GBM antibodies. The patient serum was analyzed using different assays over the years (Table 1) and none could detect circulating anti-GBM antibodies. The rate of negative results with the most commonly employed assays (ELISA) that use human or bovine substrates is about 5%. Analyses to identify the IgG subtype highlighted the presence of IgG4 types and IgG1. It has been suggested that IgG4 subclass dominance could be explained by chronic antigen stimulation. In this patient, like in many of the previously reported cases, exposure of the Goodpasture antigen might have been triggered by the chronic smoking habit. This case report highlights the need for early renal biopsy and direct immunofluorescent microscopy analysis for the diagnosis of GS in clinically suspected cases with negative serum anti-GBM antibody tests.

**Table 1:** List of the different assays used to test the patient’s serum. All were negative

2011	Enzyme-Linked Immunosorbent Assay (ELISA) with purified alpha 3 chain of the non-collagenous domain of type IV collagen as antigen.
2011 to 2017	Immunodot assay with recombinant alpha 3 chain of the non-collagenous domain of type IV collagen as antigen.
2017	Chemiluminescence assay with native alpha 3 chain of the non-collagenous domain of type IV collagen as antigen.
2017	Fluoro Enzymatic Immunoassay with recombinant alpha 3 chain of the non-collagenous domain of type IV collagen as antigen.
2017	Immunofluorescence assay with monkey tissue
2017	Multiplex particle-based flow cytometric assay with bovine native antigen.

The negative results of the serological tests can be explained by different reasons [16], particularly: (a) lack of sensitivity of the assay, especially for low-affinity antibodies [17]; (b) specific isotypes or sub-classes of anti-GBM antibodies (IgA or IgG4) that are not easily detected in ELISA or radioimmunoassays [18]; (c) disappearance of the antibodies from the circulation before the disease resolution; (d) very low levels of low-affinity antibodies due to the removal from circulation of high-affinity antibodies by an ‘immunological sink’; (e) antibodies that are highly specific for human GBM, and not for non-human (non-primate) antigens; (f) loss of lymphocyte T helpers that are required for the lymphocyte B response, resulting in the decline of auto-antibody production [19]; and (g) antibodies specific for other collagen subtypes that are not detected with the usual assays (e.g., alpha5(IV)NC1, alpha-4NC1…). The subsequent IgG subtype analysis in the transplant biopsy (showing IgG1 and a majority of IgG4) suggests the presence of a specific sub-class of anti-GBM antibodies that could not be detected by the used tests. New assays to detect rare subtypes of anti-GBM antibodies are needed.

Finally, we would like to emphasize the need of practical guidelines for patients with kidney biopsy-proven GS and seronegative anti-GBM assays to improve their follow-up and decrease the risk of GS recurrence after kidney transplantation. Indeed, the impossibility to evaluate the disease activity exposes to the risk, as illustrated in this case, of recurrence in the kidney graft. Apart from quitting smoking, which is a recognized risk factor, the usual recommendations for patients with circulating anti-GBM antibodies (delay between diagnosis and transplant, level of immunosuppression…) cannot be applied to seronegative patients.

## Abbreviations

ANCA: AntiNeutrophil Cytoplasmic Antibodies
GBM: Glomerular Basement Membrane
GS: Goodpasture Syndrome
IgG: Immunoglobulin G
MMF: Mycophenolate MoFetil
PLEX: PLasmatic EXchanges
RPGN: Rapidly Progressive GlomeruloNephritis
